# Machine learning-based predictive models and subtypes patterns in peripheral blood of schizophrenia based on a machine learning computational framework

**DOI:** 10.1038/s41537-026-00744-z

**Published:** 2026-03-24

**Authors:** Zhijun Li, Qing Sun, Haoyu Li, Naiyu Guan, Jing Ni, Jing Wang, Xiaolei Xu, Ye Shen, Siyu Sun, Yan Li

**Affiliations:** 1 Department of Epidemiology, School of Public Health, Beihua University, Jilin, China; 2 Department of Clinical Nutrition, Affiliated Hospital of Jilin Medical College, Jilin, China; 3 Laboratory of Jilin Branch, China Railway Shenyang Bureau Group Co., LTD., Shenyang Disease Prevention and Control Institute, Jilin, China

**Keywords:** Schizophrenia, Biomarkers

## Abstract

Schizophrenia (SCZ) is a complex psychiatric disorder, and its pathogenic mechanisms are not yet fully understood. The identification of reliable blood biomarkers and molecular subtypes for early diagnosis and effective therapy remains a significant challenge. To address this issue, we utilized a combination of bioinformatics and machine learning (ML) to identify potential biomarkers for SCZ. Our approach involved the integration of 12 different ML algorithms to develop a diagnostic signature based on data from several datasets, including GSE18312, GSE27383, GSE38485, GSE54913, and GSE165604. A nomogram was constructed using these datasets for potential clinical applications. In addition, clustering analysis was performed on SCZ patients using consensus clustering and non-negative matrix factorization (NMF) algorithms. We further evaluated subtype differences in biological functions and immune cells through various methods, such as gene set enrichment analysis (GSEA), gene set variation analysis (GSVA), Proteomaps, and IOBR analyses. Our results identified a diagnostic signature composed of 16 genes (APBB2, CLCN1, SYDE1, PAX5, SNAI1, DAZL, UNC93B1, PLAGL2, HS3ST1, ITPKB, PILRA, BTLA, SWAP70, AZI2, ADM, and AVPR2), which demonstrated robust performance in diagnosing SCZ across eight different datasets. A nomogram based on these genes was created, providing clinical benefits for SCZ patients. Among the identified genes, AZI2 was found to be the most critical, influencing inflammation and immunity. We also identified potential chemical compounds that could target these 16 genes. Unsupervised clustering and NMF algorithms revealed two distinct subtypes of SCZ, each associated with unique immune cell profiles, biological functions, and protein expression levels. In conclusion, this study not only developed a diagnostic signature and a novel nomogram for SCZ but also provided new insights into the subtypes of SCZ. These findings may pave the way for personalized diagnosis and treatment strategies for SCZ patients.

## Introduction

Schizophrenia (SCZ) is a severe, chronic psychological disorder. It leads to hallucinations, delusions, confusion, cognitive dysfunction, and brain damage^1,2^. SCZ can have a severe impact on potential life loss and life expectancy. More than 50% of patients experience long-term mental problems, and 20% suffer from chronic symptoms and disabilities. This is one of the major causes of disability among young patients^3,4^. The prevalence and mortality of SCZ continue to rise worldwide, causing severe economic burdens for families and society. However, prolonged therapy often provides limited efficacy^5^. To date, the exact etiology and pathogenesis of SCZ remain undetermined. It is generally believed to result from the complex interaction of genetic, neurochemical, and environmental factors. These include bacterial and viral infections, psychological, social, cultural, and economic contexts. Studies have shown that these factors play an important role in neurotransmitter, neurodevelopment, lipid metabolism, immunological diseases, and the pathogenesis of SCZ^6,7^. Current diagnosis of SCZ mainly depends on clinical manifestation standards, and the subtle early symptoms can lead to misdiagnosis, but it remains a significant challenge due to the heterogeneity of the disease and various influencing factors. In addition, the therapeutic effects of existing antipsychotic drugs are also limited and need to be improved, which often fail to address cognitive and negative symptoms adequately and induce many different kinds of side effects. Besides, previous study suggested that the biological subtyping of psychiatric syndromes might be a promising pathway for advances in drug discovery and personalized medicine, for making individual patient predictions about illness risk, treatment outcomes and illness course^8^. Therefore, it is urgent to discover and develop more effective diagnostic biomarkers and therapeutic targets for SCZ, improving diagnosis and developing targeted therapies.

Studies on the genetic mechanism and diagnostic criteria of SCZ provide the possibility for more accurate and personalized diagnosis and treatment^9^. Previous studies suggested that developing specific, peripheral-based predictive models for SCZ could contribute to the identification of blood biomarkers, given the high heritability of SCZ^10^. Personalized, predictive, and preventive medicine (PPPM/3PM) is an effective approach for predicting patient prognosis and improving treatment outcomes. It utilizes various molecular biomarkers obtained by omics studies, including early diagnostic and prognostic biomarkers, to aid clinicians to identify patients who require early intervention, improving clinical care^11–13^.

Machine learning (ML) has been demonstrated to be effective in applying robust candidate features and develop reliable risk models using clinical datasets^14,15^, which significantly complements classic experimental methods because feature selection and model choice are crucial for accurate gene essentiality prediction^16^. MLs can automatically identify hidden patterns and feature associations in high-dimensional data, and capture key molecular features and their functions and interactions of disease occurrence and development through deep mining of patient omics data, thereby offering new insights into disease mechanisms, early diagnosis, treatment response and drug development^17^. The application of ML to clinical risk modeling is on the rise, which is useful for selecting more robust candidate genes and creating optimal predictive models for the diagnosis of SCZ^14,15^. Previous studies have employed MLs on peripheral blood or brain transcriptomic data to identify numerous differentially expressed genes and proteins, and distinguish SCZ from healthy controls and stratify patient subgroups^18–20^. In addition, ML algorithms have been shown capable of predicting the treatment outcome for first-episode drug-naïve SCZ patients from the functional connection in the superior temporal cortex with an accuracy of 82.5%^21^. However, due to limited data and the heterogeneity across cohorts, low effect sizes, poor reproducibility, and low discriminative ability of models, it still lacks the research utilizing MLs for identifying potential diagnostic biomarkers of SCZ and subtyping SCZ patients. Notably, a multigene signature is likely to be a promising method for an ideal biomarker in the big data era.

Therefore, we hypothesized that bioinformatic strategy, combined with the innovative integration of multiple ML algorithms, will identify novel and effective blood diagnostic biomarkers and therapeutic targets. Our study aims to identify the peripheral diagnostic signatures of SCZ by integrating a variety of MLs from transcriptomic data, provide preliminary insights into the development of a non-invasive blood diagnostic model and the identification of molecular subtypes to deeply understand the pathogenesis of SCZ and better serve patients.

## Materials and methods

### Datasets selection and SCZ-key genes identification

Five microarray data of peripheral blood from SCZ were retrieved from Gene Expression Omnibus (GEO) database (GSE18312, GSE27383, GSE38485, GSE54913, and GSE165604). The sample sizes (cases/controls) and the number of genes analyzed for each dataset are detailed in Table S3. We separately performed data filtering and correction, log-transformation and normalization for each dataset, then merged the datasets and removed batch effect by ‘Combat’ of ‘sva’ package. The merged dataset was split into training and internal test sets (7:3 ratio). Each GEO and metadata were used for validation. Using differential expression analysis with the ‘limma’ package, we identified a significant number of differentially expressed genes (DEGs) between SCZ and control (CTL) (|LogFC | > 0.10, *P* < 0.05). Weighted gene co-expression network analysis (WGCNA) on GSE165604 revealed SCZ-related gene co-expression modules. The similarity matrix, based on Pearson correlation coefficients, was converted to an adjacency matrix with a soft threshold, then transformed into a topological overlap matrix (TOM). Hierarchical clustering identified the gene dendrogram, and Dynamic Tree Cut found co-expression modules. Module-trait association revealed key modules highly correlated with SCZ, merged into WGCNA_MEs. Finally, overlapping genes from DEGs and WGCNA_MEs were identified as SCZ-key genes. Functional enrichment analysis was performed using ‘clusterprofiler’ and Metascape^22,23^, with *P* < 0.05 considered significant.

### Establishment of the diagnostic signature and a predictive diagnosis nomogram

Using SCZ-key genes, we constructed a predictive diagnosis model with high accuracy. We developed a diagnostic signature for SCZ by combining ‘Stepglm’ and ‘RF’ among 12 ML algorithms (Enet, GBM, LDA, Lasso, Naïve Bayes, glmBoost, plsRglm, Stepglm, RF, Ridge, SVM and XGBoost)^15,24^. The best combination was chosen after extensive evaluation, with Enet, Ridge, Stepglm, and Lasso selecting key features and reducing overfitting, while SVM, LDA, GBM, glmBoost, plsRglm, Naïve Bayes, RF, and XGBoost were used for classification^15^. Areas under the receiver operating characteristic curves (AUROCs) in eight datasets (training, test, 5 GEO, meta) were evaluated and ranked, evaluations of the models’ performance were undertaken by calculating the area underneath each model’s Receiver Operating Characteristic curve (AUROC) and subsequently representing the results visually through heatmap^15,24^. To provide a comprehensive assessment, we also report the including sensitivity, specificity, Positive Predictive Value, Negative Predictive Value, precision, Recall and F1 score-for our final model(s) on both the training and all validation datasets. To assess the generalizability of our predictive model beyond the training data distribution, we employed a cross-study validation framework. After training on the combined set of five GSE datasets, the model was evaluated independently on each held-out GSE dataset. This approach provides a stringent, realistic estimate of performance on external cohorts, as it tests the model against the technical and biological heterogeneity inherent in data from independent sources, which is a key challenge in genomic biomarker translation. In addition, a predictive nomogram for SCZ diagnosis was established with the best model genes using ‘rms’ in R, offering clinical insights. The nomogram’s accuracy was assessed by ROC, Calibration curves, and DCA.

### Pathway enrichment, immune infiltration analysis and Pan-cancer analysis

We performed GSVA analyses for biological processes, immune infiltration analyses using CIBERSORT and single-sample gene set enrichment analysis (ssGSEA) algorithms evaluating the diversity of immune cells in SCZ, as well as the relationship of the diagnostic signature and immune cells by Spearman correlation analyses. Moreover, GENEMANIA, Metascape and Friendship analyses were performed to identify intersected genes and evaluate their importance^25,26^. Furthermore, Pan-cancer analysis was conducted on the crucial model gene using R package ‘TCGAplot’^27^.

### Regulation network and potential drug of diagnostic signature

We used NetworkAnalyst and Enrichr for regulation factors and potential drugs of model genes, JASPAR, MiRTarbase, and RegNetwork databases were used to establish the TFs-genes, miRNAs-genes and TFs-miRNAs co-regulatory networks. DisGeNET database was used to detect diseases related to model genes. Moreover, Comparative toxicogenomics database (CTD) and Drug Signatures Database (DSigDB) were used to investigate the chemicals and drugs associated with model genes. The data were visualized by Cytoscape software.

### Unsupervised clustering analysis and non-negative matrix factorization algorithm

After a consensus clustering approach using R package ‘ConsensusClusterPlus’, we successfully delineated two clusters within SCZ based on 12 model gene expression profiles. Then, we identified DEGs between clusters A and B through R package ‘Limma’ with |LogFC | > 0.2 and *P* values < 0.05. Subsequently, we utilized R package ‘NMF’ to conduct a clustering analysis based on the above DEGs between clusters, further uncovering possible molecular subtypes of SCZ. We utilized ‘limma’ and ‘WGCNA’ packages to identify identified the significant DEGs and key module of SCZ subgroups, and computed the interaction analysis of DEGs and key module as hub genes. CTD was used to confirm the association of hub genes with SCZ based on the Inference Score of their interaction.

### GSEA, GSVA and protein enrichment analysis and Immune infiltration

GSEA, GSVA, and protein-level enrichment analyses were conducted for model genes using R packages ‘clusterprofiler’, ‘GAVA’, ‘Limma’, and ProteoMaps tool (https://bionic-vis.biologie.uni-greifswald.de/). We analyzed DEGs of subclasses to explore potential biological processes of SCZ, and to investigate the underlying causes for these phenotypic differences^28,29^. We performed the immune infiltration analysis for SCZ subclasses using distinct algorithms of R package ‘IOBR’^30^.

## Results

### Identification of DEGs

**Fig. 1:**
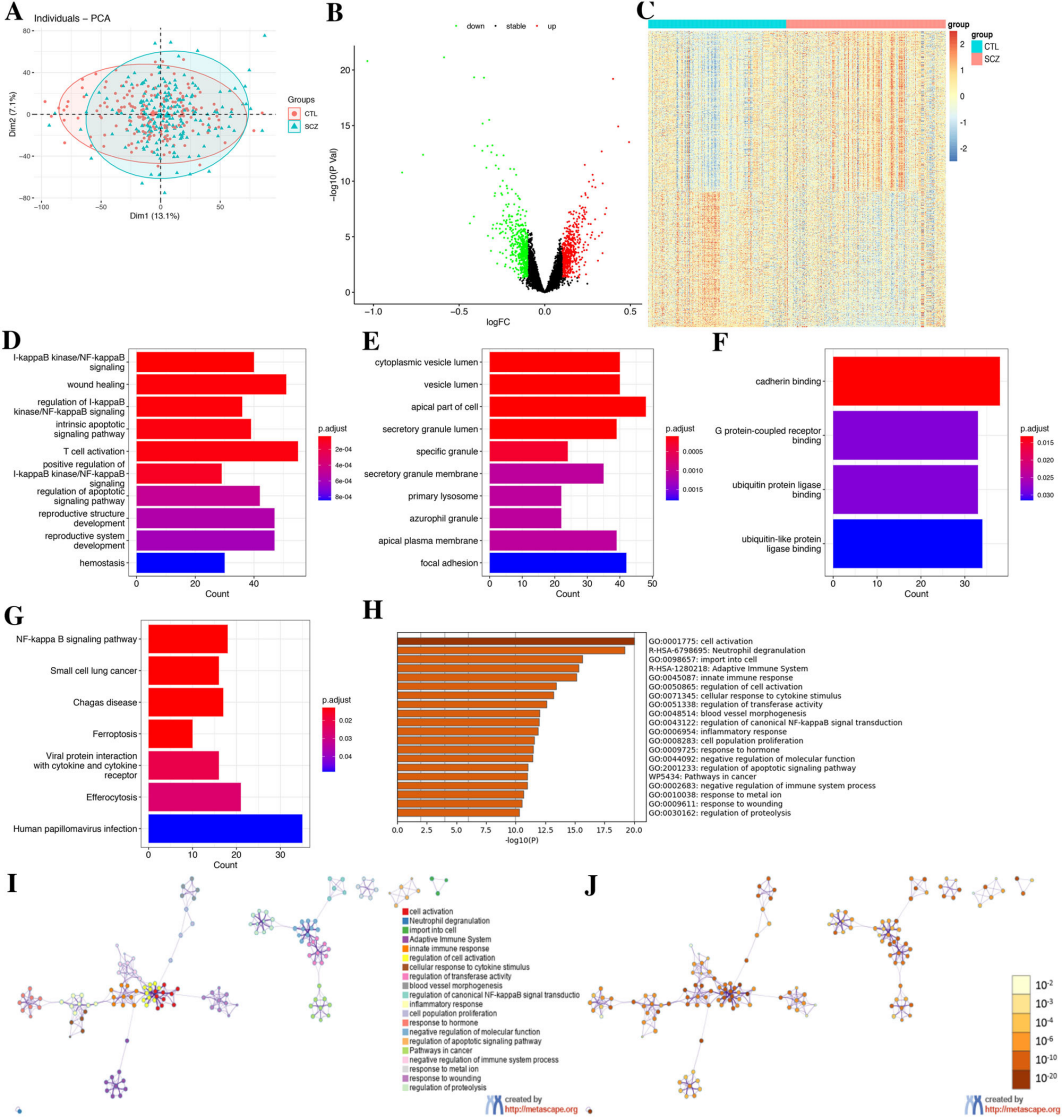
Differentially expressed genes (DEGs) and functional enrichment analysis. **A** PCA plot. **B** volcano plots of DEGs. **C** volcano plots of DEGs. **D** GO terms of biological process (BP). **E** GO terms of cellular component (CC). **F** GO terms of molecular function (MF). **G** KEGG pathways. **H** Heatmap of the top 20 enriched GO terms by Metascape. **I** Network of enriched terms colored by cluster ID, where nodes that share the same cluster ID are typically close to each other. **J** Network of enriched terms colored by *p* value, where terms containing more genes tend to have a more significant *p* value.

We identify 1039 DEGs between SCZ and CTL, with 561 upregulated and 478 downregulated genes, using a merged dataset of five blood samples (Fig. 1A–C). GO and KEGG pathway analyses showed that these DEGs were mainly enriched in I-kappaB kinase/NF-kappaB signaling, intrinsic apoptotic signaling, T-cell activation (BP); cytoplasmic vesicle lumen, apical cell part, and primary lysosome (CC); cadherin binding, G protein-coupled receptor binding, and ubiquitin protein ligase binding (MF). Additionally, they were involved in NF-kappaB signaling, ferroptosis, viral protein-cytokine/receptor interaction, and efferocytosis (Fig. 1D–G and Table S4).

Metascape analysis further indicated that DEGs participate in cell activation, innate immune response, cellular response to cytokine stimuli, regulation of canonical NF-kappaB signaling pathway, inflammatory response, and response to metal ionas. WikiPathways showed associations with cancer pathways, while Reactome involved neutrophil degranulation and adaptive immune response (Fig. 1H–J). Transcription factors identified in TRRUST database include NFKB1, RELA, SP1, TP53, EST1, AR, ETS1, STAT3, USF1, TFAP2A, OTX2, ATF4, FLI1, JUND, FOXO1, IRF1, ING4, HES1, PRARA, and USF2 (Fig. S7).

### Gene expression pattern of interested modules and SCZ-key genes identification

**Fig. 2:**
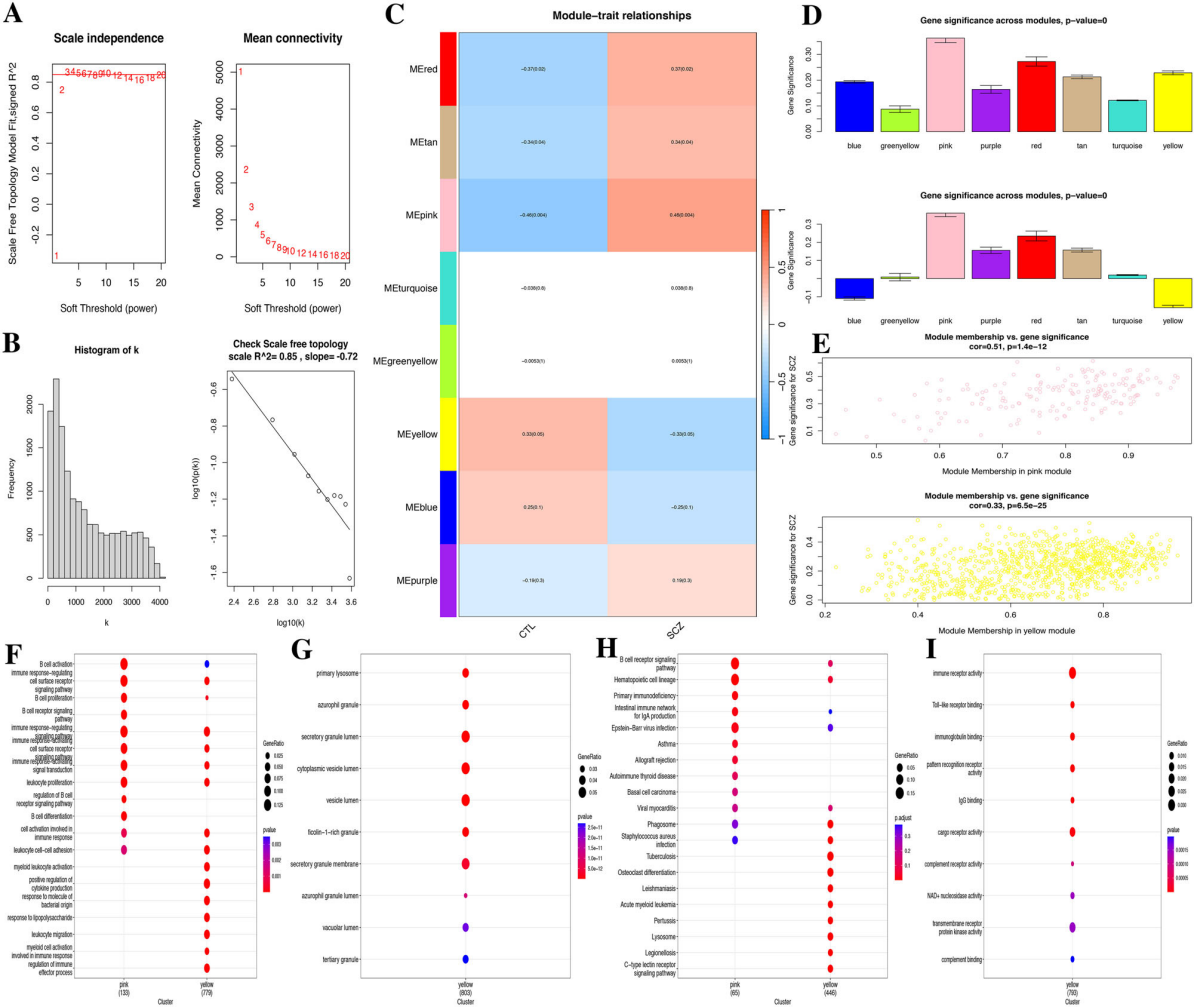
Identification of co-expressed modules and relationship of modules and disease status by WGCNA. **A** Soft threshold. **B** Free topology scale *R*^2^. **C** Correlation of modules and disease status. **D** Bar plot of mean gene significance (GS) across modules. **E** Scatterplots of GS for disease status versus module membership (MM) in four key modules. **F** GO terms of biological process (BP). **G** GO terms of cellular component (CC). **H** GO terms of molecular function (MF). **I** KEGG pathways.

At a soft threshold of *β* = 3, the scale-free *R*^2^ value reached 0.85, leading to an increase in average connectivity and the identification of eight modules (Fig. 2A–C). The pink module (169) was positively correlated with SCZ, while the yellow modules (924) were negatively correlated (Fig. 2C); Meanwhile, the correlation coefficient between gene significance (GS) for SCZ and module membership (MM) was highest for the pink module (*r* = 0.51, *P* = 1.4e-12), followed by the yellow module with a relatively high value (*r* = 0.33, *P* = 6.5e-25) (Fig. 2D). Thus, genes in the pink module were mostly over-expressed in SCZ, while those in the yellow module were mostly under-expressed. The GO and KEGG analyses results for these modules are shown in Fig. 2F–I. KEGG results indicated that the pink module was mainly involved in pathways such as B-cell receptor signaling, hematopoietic cell lineage, primary immunodeficiency, intestinal immune network for IgA production, and Epstein-Barr virus infection. In contrast, yellow modules were mainly concentrated in C-type lectin receptor signaling pathway, the B-cell receptor signaling pathway, virus myocarditis, and bacterial and viral infection (Fig. 2I). Therefore, pink and yellow modules, identified as key modules, were merged into the WGCNA_MEs, resulting in the identification of 249 key genes (Fig. 2E and Supplementary Table S5).

Furthermore, we performed the intersection analysis of DEGs and WGCNA_MEs to further delve into the pathogenesis of SCZ, identifying 78 overlapping genes as SCZ-key genes (Fig. S1A and Table S6). These 78 genes are associated with inflammatory responses, positive regulation of cell migration and apoptotic cell activation, astrocyte development, viral protein interactions with cytokines and cytokine receptors, leukocyte migration, innate immune responses, amide transport, and cellular response to lipid. Reactome analysis revealed involvement in neutrophil degranulation, iron uptake and transport, and GPCR ligand binding (Fig. S1B, C).

In the COVID database, 45 terms were identified, with the top five being RNA expression changes in CD14+ monocytes from patients at various stages of disease severity (Fig. S1D). In the DisGeNET database, the top terms associated with the identified genes were infection, adult diffuse large B-cell lymphoma, acute promyelocytic leukemia, HIV-1 infection, and lung disease (Fig. S1E).

The GO analysis heatmap in PaGenBase showed that the identified targets were tissue-specific to blood and spleen, cell-specific to CD14+ monocytes, and tissue-specific to bone marrow (Fig. S1G). Additionally, transcription factors identified in the TRRUST database included NFKB1, RELA, SP1, and CREB1 (Fig. S1H).

### Construction and assessment of the diagnostic signature and a predictive nomogram

After comprehensive screening of the 133 combinations, we identified Stepglm[both]+RF and Stepglm[backward]+RF as the final predictive models for diagnosing SCZ (Fig. S2A and Table S7). The AUROCs in eight sets (Train, Test, Meta, GSE18312, GSE27383, GSE38485, GSE54913, GSE165604) were 1.000, 0.913, 0.986, 0.856, 0.980, 0.999, 0.972, and 0.786, respectively (Fig. S2A and Table S7). These models consist of the same sixteen feature genes: *APBB2*, *CLCN1*, *SYDE1*, *PAX5*, *SNAI1*, *DAZL*, *UNC93B1*, *PLAGL2*, *HS3ST1*, *ITPKB*, *PILRA*, *BTLA*, *SWAP70*, *AZI2*, *ADM*, and *AVPR2* (Table S8).

Furthermore, we developed a predictive nomogram using these 16 genes. The AUROCs in the eight datasets were 0.886, 0.871, 0.864, 1.000, 0.917, 1.000, 1.000, and 1.000, respectively (Fig. S2R). This nomogram demonstrated high predictive accuracy and provided a better net clinical benefit, as indicated by the calibration curves and DCAs in the eight datasets (Fig. S2B–Q). Thus, we have identified a final predictive model with high accuracy and translation relevance for diagnosing SCZ, and the predictive nomogram provided a robust and accurate clinical application tool.

### Enrichment patterns for diagnosis signature and Pan-cancer analysis

**Fig. 3:**
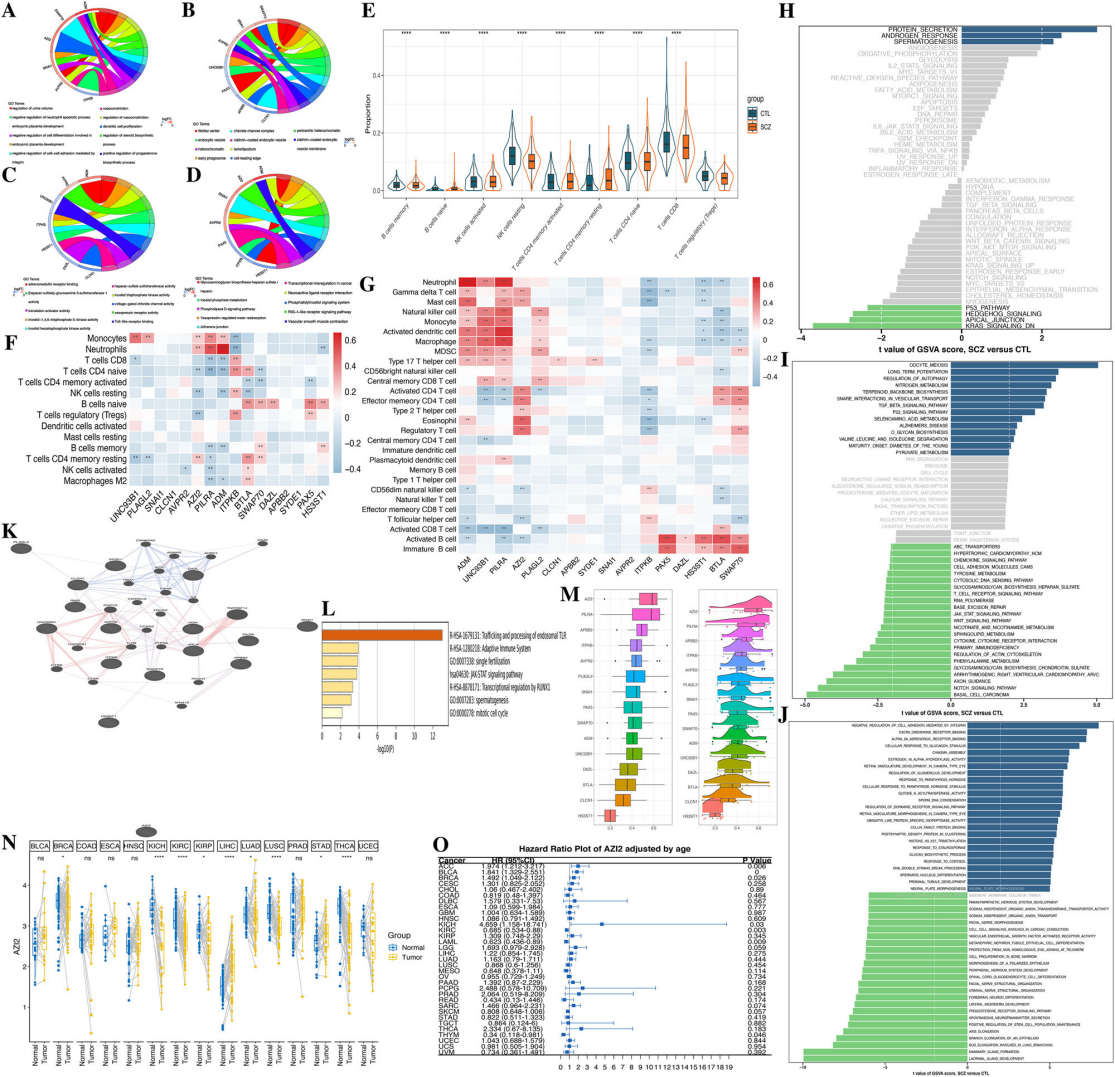
Model gene enrichment patterns, Immune Landscape and Pan-caner analysis. **A** Chords plot of biological process (BP). **B** Chords plot of cellular component (CC). **C** Chords plot of molecular function (MF). **D** Chords plot of KEGG pathways. **E** The proportion of immune cells between brain tissues in SCZ and normal control. **F** Correlation between key genes and immune cells by CIBERSORT algorithm. **G** Correlation between key genes and immune cells by ssGSEA algorithm. **H**–**J** Enrichment patterns of SCZ by GSVA enrichment analysis with KEGG pathways (**H**), HALLMARK (**I**), and biological process gene sets (**J**). **K** PPI network map by GENEMANIA. **L** Biological pathway enrichment map. **M** Friendship analysis. **N** Pan-cancer differential analysis. **O** Pan-caner survival analysis. *P < 0.05, **P < 0.01, ***P < 0.001.

To some extent, the aforementioned findings suggest that the 16 model genes may play significant roles in SCZ pathogenesis. In this study, expressions of *SNAI1*, *BTLA*, *SWAP70*, *AZI2*, *ADM* and *AVPR2* were upregulated in SCZ; whereas *APBB2*, *CLCN1*, *SYDE1*, *PAX5*, *DAZL*, *UNC93B1*, *PLAGL2*, *HS3ST1*, *ITPKB* and *PILRA* were downregulated. The GO and KEGG results for these sixteen model genes are presented in Fig. 3A–D and Table S9.

GSVA analysis revealed that the upregulation of *SNAI1*, *SWAP70*, *AZI2*, and *ADM*, along with the downregulation of *PAX5*, *DAZL*, *HS3ST1*, and *ITPKB*, were implicated in various pathways such as protein secretion, androgen response, MTORC1 signaling, complement, TNFA signaling via NFKB, apoptosis, inflammatory response, peroxisome, IL6-JAK-STAT3 signaling, G2M checkpoint, fatty acid metabolism, IL2-STAT5 signaling, E2F targets, interferon gamma response, KARS signaling up, and TGF-BETA signaling. Additionally, the upregulation of *BTLA*, *AVPR2*, and the downregulation of *UNC93B1*, *PLAGL2*, and *PILRA* were associated with epithelial-mesenchymal transition, estrogen response early, DNA repair, apical junction, MYC targets v2, myogenesis, and KRAS signaling DN. The downregulation of *APBB2*, *CLCN1*, and *SYDE1* was enriched in processes such as SNARE interactions in vesicular transport, TGF-BETA signaling pathway, regulation of autophagy, and MTOR signaling pathway (Fig. S3).

Furthermore, an in-depth exploration of the biological significance of these sixteen model genes was conducted. Twenty genes were closely associated with these 16 genes, which were significantly enriched in trafficking and processing of endosomal TLR, adaptive immune system, single fertilization, JAK-STAT signaling pathway, transcriptional regulation by RUNX1, spermatogenesis, and mitotic cell cycle (Fig. 3K, L). *AZI2* emerged as the most important gene in the diagnostic signature, contrasting with *HS3ST1* expression but showing synergy with other model genes (Fig. 3M).

Given the role of AZI2 in innate immune signaling and the increasing recognition of immune dysregulation as a common feature across diverse diseases, we sought to explore its potential broader relevance. We analyzed AZI2 expression correlation with key immune signatures across 33 cancer types from The Cancer Genome Atlas (TCGA) to determine if its immune-associated role observed in our primary analysis is recapitulated in an oncological context, where immune dysfunction is a well-established driver (Fig. S8). Pan-cancer analysis of *AZI2* showed significant upregulation in LIHC and STAD, and significantly downregulated in BRCA, KICH, KIRC, KIRP, LUAD, and LUSC (Fig. 3N). Cox regression analysis for OS indicated that *AZI2* acted as a risk factor in ACC, BLCA, BRCA, KICH, and THCA, but as a protective factor in KIRC and LAML (Fig. 3O). The results of TMB, MSI, and correlation analyses with immune features and immune cells of *AZI2* are presented in Fig. S8.

### Enrichment patterns in SCZ

The GSVA results of SCZ indicated significant upregulation in several pathways, including oocyte meiosis, long-term potentiation, autophagy regulation, nitrogen metabolism, terpenoid backbone biosynthesis, SNARE interactions in vesicular transport, TNF-beta signaling, P53 signaling, selenoamino acid metabolism, Alzheimer’s disease, valine leucine, and isoleucine degradation, maturity-onset diabetes of the young, and pyruvate metabolism (Fig. 3H–J).

Additionally, CIBERSORT immune infiltration analysis showed upregulation of monocytes, resting memory CD4 + T cells naïve CD4 + T cells in SCZ, with a downregulation trend observed in memory B cells, B naïve cells, activated NK cells, CD8 + T cells, and regulatory T cells (Tregs) (Fig. 3E). Correlation analysis revealed significant positive correlations between ADM, UNC93B1, and PIKRA, gamma delta T cells, mast cells, NK cells, monocyte, activated dendritic cells, macrophage, MDSCs, and Type 17 T helper cells. Conversely, ITPKB, DAZL, HS3ST1, and BTLA were negatively correlated with these immune cells. *PAX5*, *HS3ST1*, *BTLA*, and *SWAP70* were positively correlated with activated and immature B cells (Fig. 3F, G).

### Translation regulators identification and drugs prediction

Fifty-five TFs and 551 miRNAs were identified (Fig. S9A, B). The top 5 TFs were FOXC1, RELA, NFIC, IRF2, and SREBF1, while the top 5 miRNAs were miR-335-5p, miR-93-5p, miR-26b-5p, miR-6884-5p, and miR-47566-5p (Fig. S9A, B). According to the TF-miRNA co-regulatory network (Fig. S9C), the top 5 model genes were *PAX5*, *PLAGL2*, *APBB2*, *ADM*, and *SYDE1*. These genes were regulated by the top five TFs-CTCF, NKX2-2, FOXO4, USF1, and SRF, which cooperated with miRNAs such as miR-200c, miR-30-a, miR-141, miR-571, and miR-410. Based on the connectivity between miRNAs, genes, and TFs, combinations such as miR-200c-PAX5-CTCF, miR-200c-PAX5-NKX2-2, miR-200c-PAX5- FOXO4, miR-200c-APBB2-CTCF, miR-30a-PLAGL2-MYC, and miR-410-PLAGL2-EGR1 have potential as biomarkers for the diagnosing and treating SCZ.

The DisGeNET database revealed the most highly correlated genetic diseases, including Liver Cirrhosis, Chemical and Drug-Induced Liver Injury, Autistic Disorder, Bipolar Disorder, Glomerulonephritis, Hyperalgesia, Pain, SCZ, Gastric Ulcer, Sepsis, Herpes Encephalitis, Encephalitis, Myelitis and Encephalomyelitis in viral diseases classified elsewhere (Fig. S4A, B). Using the CTD database, major chemicals associated with the model genes were identified as Valproic Acid, 4-(5-benzo[1,3]dioxol-5-yl-4-pyridin-2-yl-1H-imidazol-2-yl)benzamide, (6-(4-(2-piperidin-1-ylethoxy)phenyl))-3-pyridin-4-ylpyrazolo[1,5-a]pyrimidine, Trichostatin A (Fig. S4C and Table SI). Meanwhile, the top 10 drugs were abstracted from the DSigDB database based on P-value (Fig. S4D and Table S2), provinding novel therapeutic options for treating SCZ.

### Classification patterns of SCZ in blood expression profilings

**Fig. 4:**
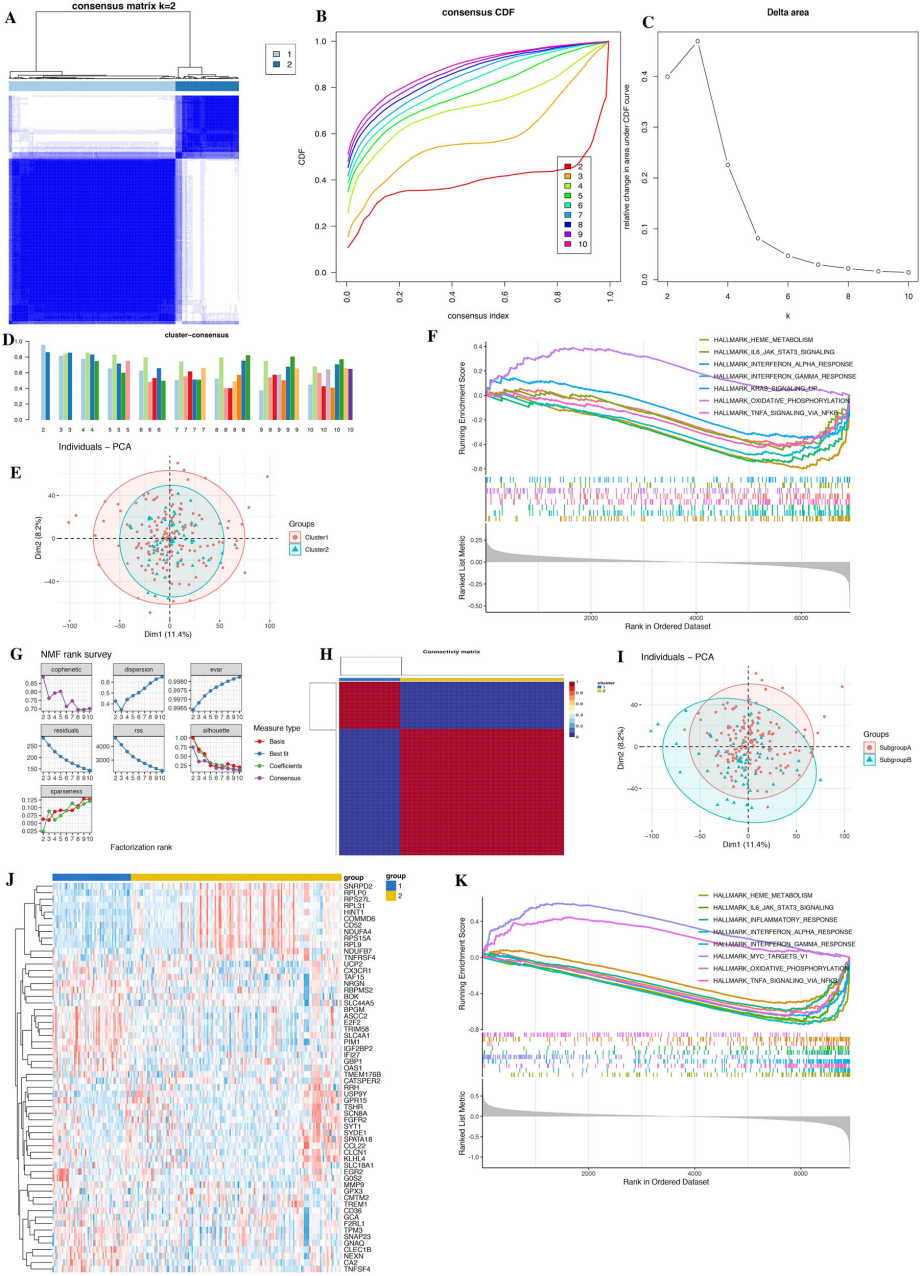
Unsupervised clustering analysis and Non-negative matrix factorization algorithm. **A** Consensus clustering matrix for *k* = 2, defining two different subtypes. **B** Cumulative distribution function (CDF) curves for *k* = 2–10. **C** CDF delta area curves. **D** Consensus clustering scores when *k* is 2–10. **E** Principal component analysis (PCA) visualization of the sample distribution of the two clusters. **F** Gene set enrichment analysis (GSEA) between two clusters. **G** Distribution of cophenetic, residuals, RSS, silhouette, Var and dispersion with a rank of 2–10. **H** Heatmap of NMF clustering at *k* = 2. **I** PCA plots showing differences in sample clustering between two SCZ gene subgroups. **J** Heatmap of key genes in NMF clustering. **K** Gene set enrichment analysis (GSEA) between two subgroups.

Consensus clustering analysis, based on 16 model genes, accurately divided 214 SCZ samples into two clusters: Cluster 1 (*n* = 155) and Cluster 2 (*n* = 59) (Fig. 4A–E). GSEA analysis identified 79 differentially DEGs between the clusters, mainly involved in NOD-like receptor signaling, cytokine-cytokine receptor interactions, Parkinson’s disease, and ribosome pathways (Fig. 4F and Table S10). Furthermore, NMF analysis, using these 79 DEGs, successfully divided the 214 SCZ patients into two subgroups: Subgroup 1 (*n* = 156) and Subgroup 2 (n = 58) (Fig. 4G–J). We found 137 DEGs between these subgroups, with GSEA analysis results shown in Fig. 4K and Table S10.

With a soft threshold of 4, 22 modules were identified, with the red and lightgreen modules positively correlated with Subgroup A. These two modules contained 41 key genes (Fig. S5 and Table S11). By intersecting the subgroup DEGs with the key module genes, we obtained 34 hub genes (Fig. S5 and Table S11). Analysis using the Reactome database revealed that these hub genes are involved in platelet activation, signaling, and aggregation, as well as translation. GO biological processes include blood coagulation and positive regulation of platelet activation. CORUM analysis highlighted the ITGA2b-ITGB3-CD9-GP1b-CD47 complex, while KEGG pathways associated with serotonergic synapse and COVID-19 were identified using Metascape. Finally, the CTD database confirmed the involvement of these hub genes in the progression of SCZ or other mental disorders.

### GSVA and protein enrichment analysis, and immune infiltration of subtypes

Based on KEGG pathways, GSVA results indicated significant enrichment of metabolic processes in Cluster 1and Subgroup 1. Conversely, inflammation and immune processes were enriched in Cluster 2 and Subgroup 2 (Fig. S6A–F). Proteomap results further indicated a high similarity in protein enrichment patterns between these Clusters and Subgroups (Fig. S6H–K). Immune infiltration analyses, using six algorithms, were also presented for SCZ Subtypes in Fig. S6G. Overall, two distinct patterns were revealed associated with different biological functions in SCZ, highlighting the important role of model genes in regulating metabolic processes and the immune system.

## Discussion

With the increasing prevalence and severe impacts of SCZ, its underlying mechanism remains unclear. Identifying suitable SCZ subtypes and powerful biomarkers is necessary for early diagnosis and therapy. Although previous studies have proposed potential diagnostic biomarkers or underlying gene expression patterns for SCZ utilized transcriptomics data and MLs, none have achieved clinical practice due to the complexity of the disease, heterogeneity across limited datasets, and low discriminative ability of diagnosis models^20,31^. It’s urgent to discover the robust biomarkers specific to SCZ’s multifactorial pathology, while blood biomarkers for SCZ may be feasible^32^, which still remains room for further refinement. In this study, we performed an integrated bioinformatics and ML algorithms for mining hidden patterns and complex interactions of SCZ by processing large-scale data, and identifying potential blood biomarkers and targets for SCZ. Firstly, we identified 78 key SCZ-related genes through intersection analysis of DEGs with results from a WGCNA of SCZ, which are mainly involved in inflammatory and immune response, amide transport, and cellular response to lipid. These findings resonate with the previous results of bulk or single-nucleus RNA sequencing studies to identify significant transcriptional changes in immune response and neurodevelopmental pathways across various cell types in the blood and brain of SCZ patients^20,32,33^. Secondly, we developed a 16-gene predictive diagnostic signature with high accuracy and translatability using integrated ML algorithms. Additionally, we constructed a nomogram incorporating these signature genes, which demonstrated commendable performance by improving characterization for patients with SCZ. Previous studies have used SVM-REF, random forest, and least absolute shrinkage and selection operator (LASSO) algorithms to identify hub genes developing diagnostic nomogram models for SCZ based on ferroptosis^34^ or oxidative stress-related hub genes^31^ or differentially methylated genes (DMGs)^35,36^. However, the solitary use of ML, the uniqueness and inappropriateness of selected modeling methods and the lack of strict validation in large multicenter datasets limited their wide application in clinical settings and needed further research. In addition, the cross-combination of feature selection of ML algorithms, when they converge on certain genes, indicates a stronger consensus regarding the importance of these genes^37^. Our study used the innovative integration of multiple MLs to identify more robust peripheral diagnostic biomarkers and uncover the underlying mechanism of SCZ, offering a novel approach with a non-invasive diagnostic model to better serve patients with SCZ and complements previous research. Thirdly, we distinguished two classification patterns in the blood of SCZ patients through consensus clustering analysis. Furthermore, we used the non-negative matrix factorization (NMF) algorithm to subclassify these patterns based on DEGs, revealing significant differences in immune infiltration and diverse biological functions, including protein levels, among different subtypes. Previous studies have developed the distinct subtypes of SCZ based on blood DNA methylation profiles or Structural brain networks with gray matter volume reductions using NMF or k-means clustering^38–40^. Developing SCZ subtype patterns using blood transcriptomic data offers a novel approach to better understand the heterogeneity among SCZ patients, thereby adopting appropriate intervention measures to slow down SCZ development and complements previous research.

The predictive diagnostic signature for SCZ was constructed using Stepglm with Random Forest (RF) algorithms and comprised sixteen genes: *APBB2*, *CLCN1*, *SYDE1*, *PAX5*, *SNAI1*, *DAZL*, *UNC93B1*, *PLAGL2*, *HS3ST1*, *ITPKB*, *PILRA*, *BTLA*, *SWAP70*, *AZI2*, *ADM*, and *AVPR2*. A key finding of our validation was the range in model performance (AUC: 0.78-0.99) across the five independent GSE datasets. Even through our model maintains a favorable balance in the comprehensive performance metrics, while this variation is informative rather than merely incidental. It underscores the substantial impact of technical batch effects, platform differences, and cohort-specific clinical characteristics on the portability of genomic signatures. This analysis directly informs the contexts in which our model is most immediately reliable and where further refinement is needed. Further analysis revealed *AZI2* as the most significant gene in SCZ, significantly upregulated in blood. It is implicated in multiple biological processes, including protein secretion, androgen response, MTORC1 signaling, complement activation, TNFA signaling via NFKB, apoptosis, inflammatory response, peroxisome function, IL6-JAK-STAT3 signaling, G2M checkpoint regulation, fatty acid metabolism, IL2-STAT5 signaling, E2F targets, interferon gamma response, KARS signaling, and TGF-BETA signaling. These processes are closely associated with inflammation regulation, the immune system, and fatty acid metabolism. Our findings were consistent with the existing researches that the pathophysiology of SCZ is associated with the inflammatory, immune system and metabolism system, including innate immune activation, proinflammatory cytokine secretion, T-cell activation, and autoantibody production^41,42^. In addition, the most distinct changes of plasma metabolism in adolescents were observed in SCZ patients among three psychiatric disorders (MDD, BPD and SCZ), and the unique characteristics of significantly altered fatty acid, glycolysis, glycerophospholipid, and sphingolipid metabolism in SCZ^43^. Our findings advanced the understanding of the molecular etiology of SCZ and provided new clues regarding molecular mechanisms and treatment therapy in SCZ, and need further research.

Notably, *AZI2* is associated with immune infiltration in SCZ, with its expression levels influencing various immune cells. Specifically, *AZI2* positively correlates with regulatory T cells, eosinophils, type 2 T helper cells, effector memory CD4 T cells, activated CD4 T cells, mast cells, and gamma delta T cells, while negatively correlating with activated B cells, T follicular helper cells, and CD56dim NK cells. Furthermore, 12 transcription factors (TFs) and sixteen miRNAs that bound to *AZI2* were identified, suggesting that high *AZI2* expression in SCZ may promote inflammation and an immune response progression by enhancing immune infiltration and post-transcriptional regulation. This finding provides further insight into the specific mechanisms underlying SCZ. Besides, the *AZI2* 3’UTR acts as a novel, independent transcriptional regulator of SLC6A3, which is associated with an epistatic protective effect against substance use disorders (SUDs)^44^.

SCZ patients have a higher mortality rate from cancers. *AZI2* also plays a role in cancer^45,46^, as indicated by pan-cancer analysis showing it acts as a risk factor in several types of cancer while being protective in others^47^. Additionally, *AZI2* is involved in the *AZI2*-TBK1-IFN signaling pathway^48^, which can make cancer cells more immunogenic^49–51^. The 3p24.1 locus, related to the risk of diffuse large B-cell lymphoma, interacts with the *AZI2* promoter^52^. An unexpected but informative finding was the consistent correlation of AZI2 with immune cell infiltration across multiple cancer types (Fig. S8). While our study focuses on SCZ, this pan-cancer analysis suggests that AZI2 may function as a more general regulator of immune cell activity or recruitment within diseased tissue microenvironments. This observation supports the biological plausibility of our findings in SCZ, as it positions AZI2 within a conserved pathway of immune-tissue interaction that can be perturbed in both neuropsychiatric and oncological contexts. It also raises intriguing questions about whether therapies targeting such shared immune pathways could have trans-disease relevance.

In addition to *AZI2*, other genes in the signature also exhibit significant associations with SCZ and related conditions. For example, *APBB2* is associated with SCZ and cognitive ability in children^53^. *DAZL* is significant in SCZ with gender-specific differences^54^. *ADM*, upregulated in SCZ patients, may be a susceptibility factor and contribute to SCZ pathology^55^. *PAX5* and *ITPKB* are candidate risk genes for autism spectrum disorder (ASD) and neurodevelopmental abnormalities^56,57^. *UNC93B1* plays a critical role in antiviral innate immunity and has therapeutic potential in systemic lupus erythematosus (SLE)^58^. *PLAGL2* is associated with the development of malignancies. *HS3ST1* is associated with working memory in probable-MCI patients^59^. *PILRA* was a potential novel prognosis biomarker and therapeutic target for cancer immunotherapy^60^. A GWAS study revealed SNPs of BTLA genes for mood and anxiety disorders^61^. *SWAP70* plays an important role in immune cell maturation, and cell transformation^62^. *AVPR2* mutations identification can facilitate early diagnosis of nephrogenic diabetes insipidus (NDI)^63^.

Current evidence indicates that immune and inflammatory activation significantly impact SCZ development. We performed quantification analysis of 28 immune cell infiltration scores utilizing the ssGSEA analysis, and showed the correlations between gene expression and immune cells in SCZ. Monocytes, resting memory CD4 + T cells, and naïve CD4 + T cells were upregulated in SCZ, while memory B cells, naive B cells, activated NK cells, CD8 + T cells, and regulatory T cells (Tregs) exhibited the opposite trend. A recent comprehensive meta-analysis on the immune cell alteration in psychotic disorder showed that a broad activation of the immune system in SCZ, with cells from both the myeloid and the lymphoid line being elevated, which resonates with our findings that may provide new forms of treatment and diagnostic parameters centered around the immune system^64^. In addition, *ADM*, *UNC93B1*, and *PIKRA* were positively correlated with certain immune cells, while *ITPKB*, *DAZL*, *HS3ST1*, and *BTLA* were negatively correlated.

Identifying biologically distinct patient subgroups within common psychiatric syndromes based on the resolving illness heterogeneity provide hope for enhancing the understanding of biological alterations of diseases, and for advancing in drug discovery and personalized medicine, which will shift towards altering specific targeted biological processes in drug development and pharmacological interventions, not the complex behavioral features^8^. Unsupervised clustering analysis based on model genes effectively classified SCZ patients into two subgroups with distinct metabolic and inflammatory profiles. GSVA revealed that one subgroup was mainly associated with metabolic pathways and processes, while the other was mainly associated with the inflammatory response and the immune system. Prior research has revealed potential metabolite features of SCZ, including reduced levels of essential polyunsaturated fatty acids and vitamin E, and elevated levels of lipid peroxidation metabolites and glutamate^43,65^. In addition, the immune system and inflammation also play critical roles in SCZ pathophysiology. These findings suggest that the heterogeneity among SCZ patients and different subgroups may benefit from different interventions and treatments. Previous studies have identified subgroups with different patterns of biological features based on neuroimaging and peripheral immune markers that provide translational targets for novel drug discovery, which, combined with psychological and social perspectives in the longer term, may improve the precision with which pharmacological interventions^8,66^. Especially for identifying biomarkers at onset and specifying the progression over the early course of SCZ, studies of antipsychotic-naïve first-episode SCZ in China, including data-driven approaches, are helping identify candidate biomarkers related to early-stage illness, treatment effects, and biological subgroup differentiation. Chinese researchers are always making contributions to precision psychiatry in SCZ^66^. Further study is needed to translate these biomarkers for clinical application and to improve the validity and reliability of biologically derived subtypes, and better characterize their clinical, developmental and psychosocial features.

There are several limitations that provide context for the results and direction for future research. First, our analysis for the development and validation of diagnostic signature and the nomogram model is based solely on publicly available transcriptomic data, and further validation with more datasets is needed to ensure generalizability. Additionally, more clinical follow-up and large-sample researches are required to validate the findings. In addition, while we performed rigorous cross-dataset validation, experimental validation using qRT-PCR or protein-based assays in independent, prospectively collected cohorts is necessary to confirm the clinical utility of the identified biomarker genes. Second, we integrated datasets from different but related biological sources (peripheral blood, PBMCs, lymphocytes). Although we applied advanced batch-effect correction methods to minimize technical and source-specific variation, the inherent differences in cellular composition and transcriptional profiles across these sources represent a layer of biological heterogeneity that may influence our integrated signals. Consequently, our model likely captures a conserved, systemic immune response rather than a precise cell-type-specific signature. Future studies employing single-cell RNA sequencing or flow cytometry on uniformly processed samples are needed to refine these biomarkers and attribute them to specific immune cell subsets in SCZ.

## Conclusion

In this study, we performed an integrative meta-analysis of multiple blood-based transcriptomic datasets to develop a robust, 16-gene diagnostic signature for SCZ. Our predictive model demonstrated strong and generalizable performance across independent cohorts, validating its reliability. Furthermore, by applying consensus clustering and NMF to this signature, we identified two biologically distinct SCZ subclasses characterized by divergent immune and metabolic pathways, suggesting that the clinical heterogeneity of SCZ may arise from distinct underlying molecular mechanisms. The findings provide a peripheral biomarker candidate and a methodological pathway for subclass discovery in complex SCZ, which present new targets for mechanistic exploration and drug discovery. Future studies should confirm in larger, longitudinal cohorts and investigate their correlation with symptoms, cognitive profiles, and treatment outcomes.

## Supplementary information

Figure S1. 78 overlapping genes and functional enrichment analysis

Figure S2. A diagnostic signature of SCZ was developed.

Figure S3. Enrichment pathway analysis.

Figure S4. Regulation factors of feature genes.

Figure S5. Identification of hub genes between the two subgroups by the DEGs and WGCNA method.

Figure S6. Biological function and pathway, immune landscape and protein-level differences between the two subtypes.

Figure S7. Functional enrichment analysis by Metascape.

Figure S8. Pan-cancer analysis of AZI2.

Figure S9. Regulation factors.

Supplemental Table 1–11

Supplemental files legends

## Data Availability

The gene expression datasets (GSE18312, GSE27383, GSE38485, GSE54913, and GSE165604) analyzed in this study are publicly available in the NCBI GEO repository. The data underlying this article are available in the article and in its online supplementary material. All code generated or used during the study is available from the corresponding author by request.
