# Supplementary figures and images for: Machine learning-based predictive models and subtypes patterns in peripheral blood of schizophrenia based on a machine learning computational framework
Source: Schizophrenia (Heidelb). 2026 Mar 24;12(1):46. doi: 10.1038/s41537-026-00744-z (PMC13187130; doi:10.1038/s41537-026-00744-z)

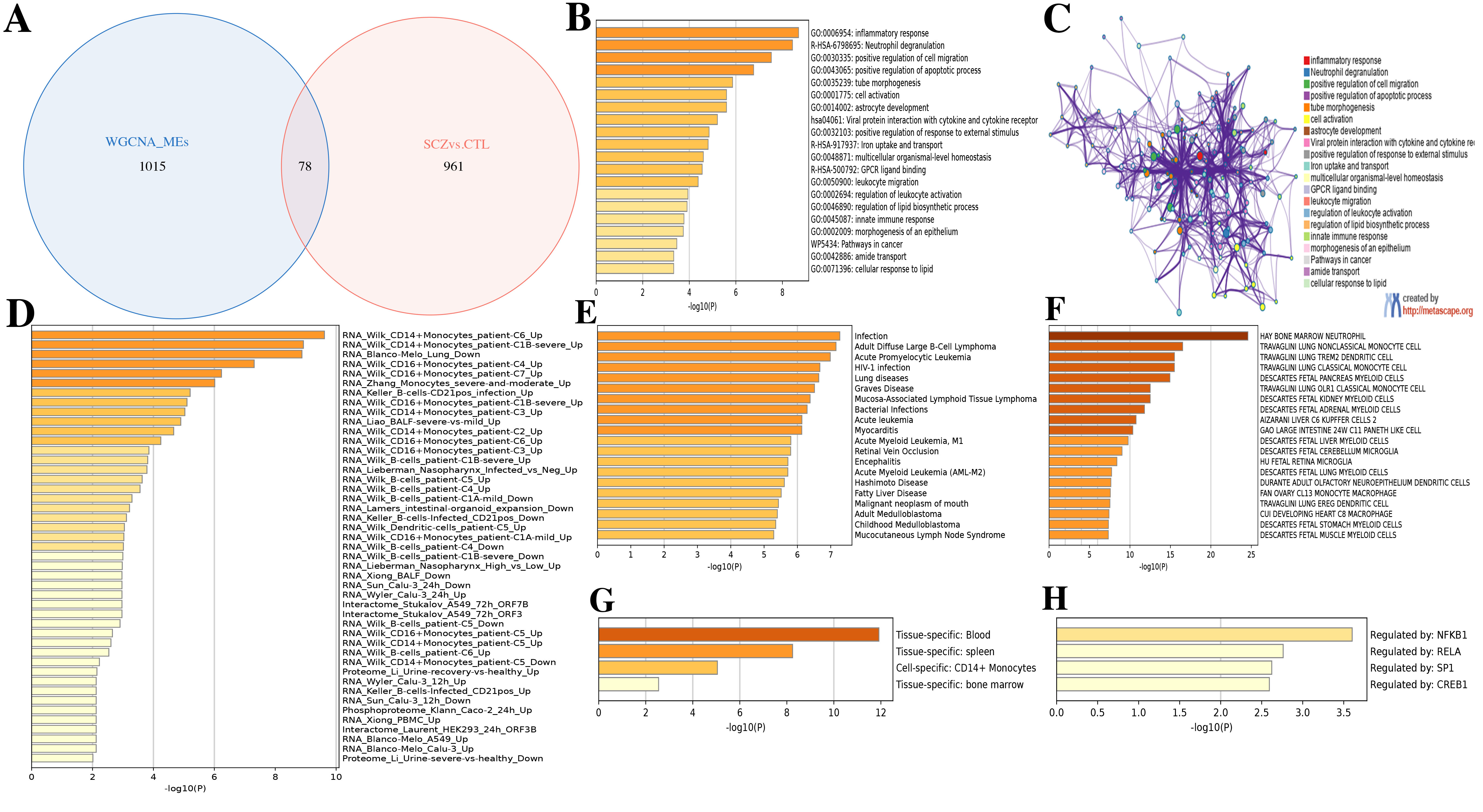

Supplement: Supplementary file 1 — Figure S1. 78 overlapping genes and functional enrichment analysis [file 41537_2026_744_MOESM1_ESM.jpg]

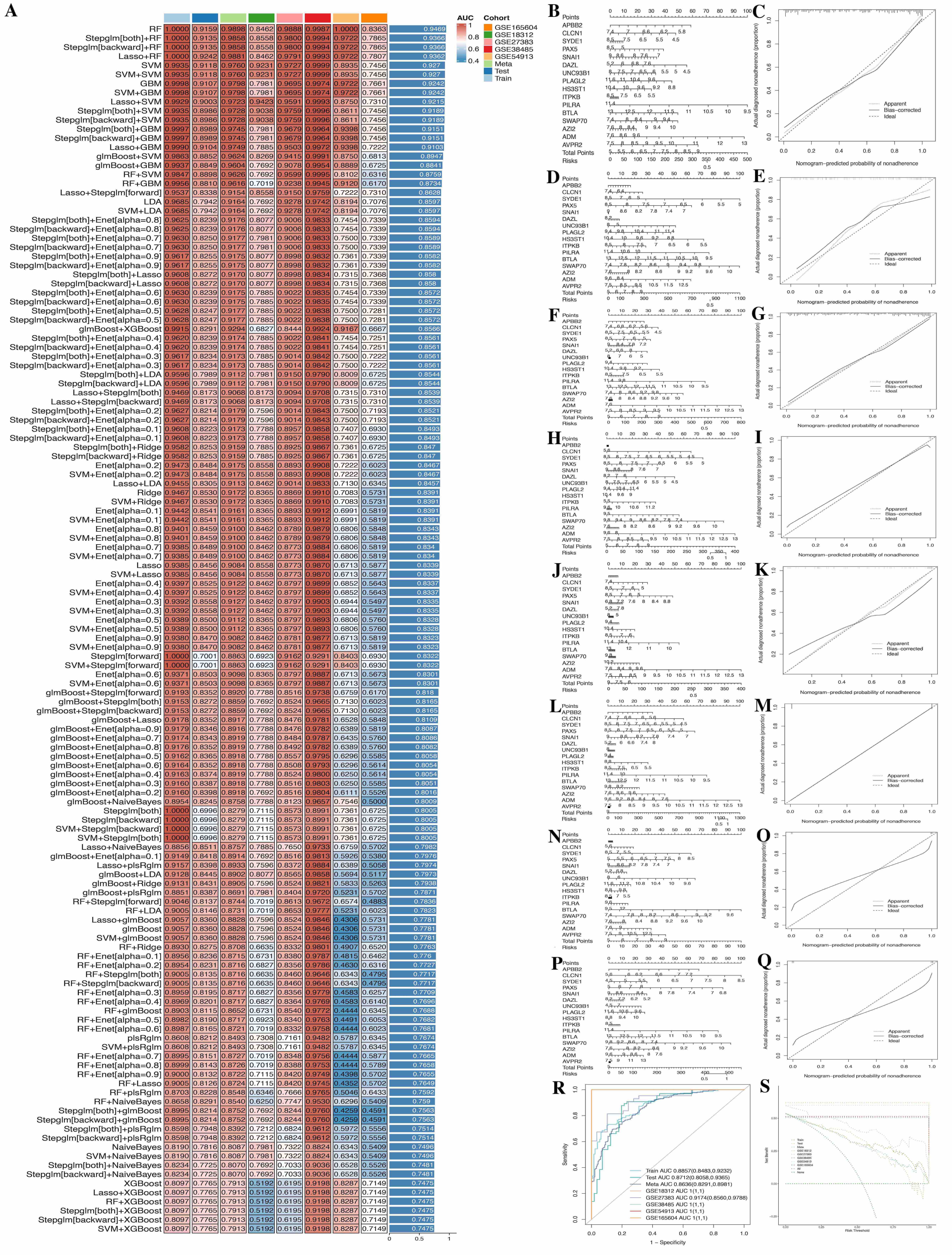

Supplement: Supplementary file 2 — Figure S2. A diagnostic signature of SCZ was developed. [file 41537_2026_744_MOESM2_ESM.jpg]

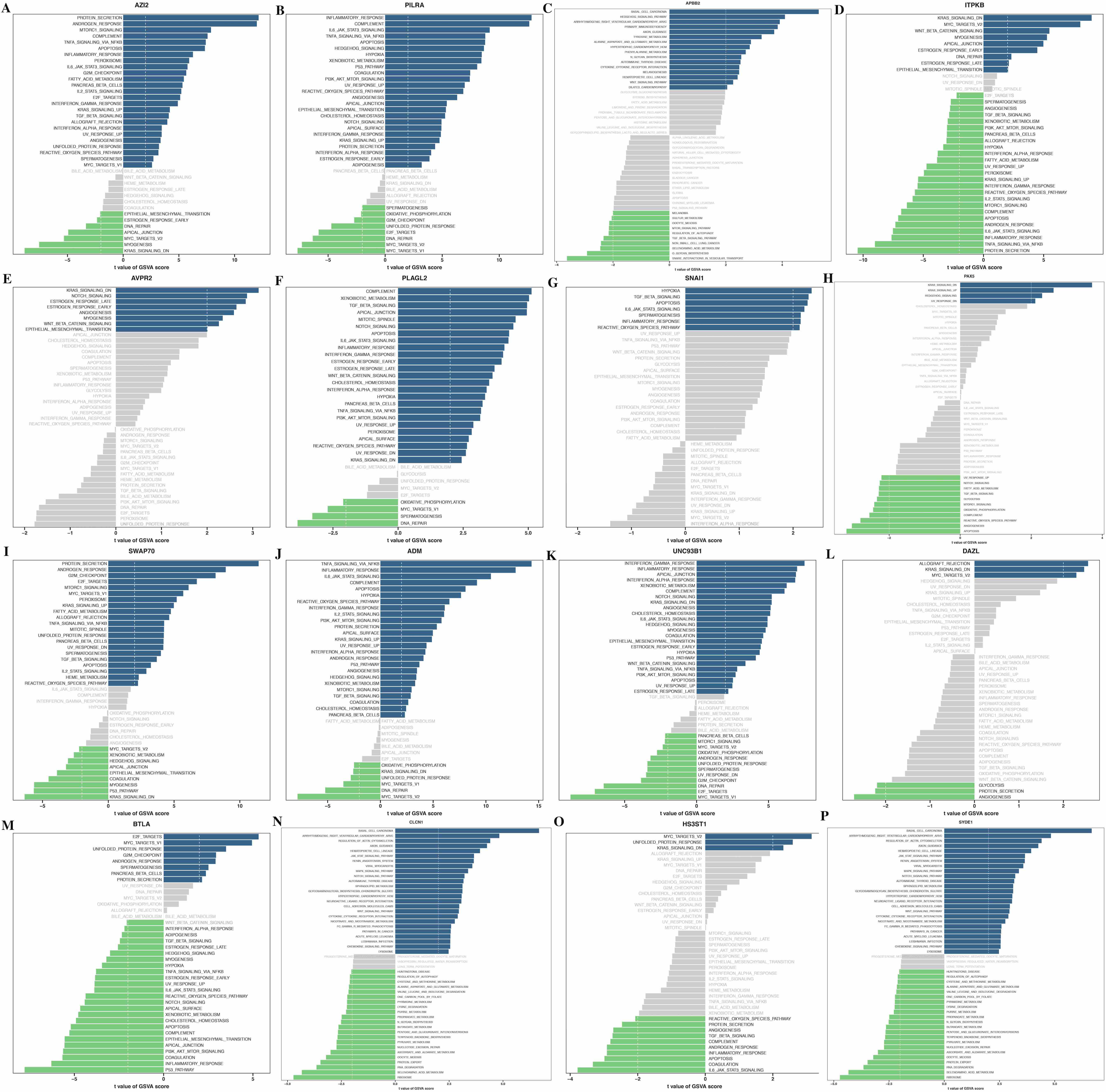

Supplement: Supplementary file 3 — Figure S3. Enrichment pathway analysis. [file 41537_2026_744_MOESM3_ESM.jpg]

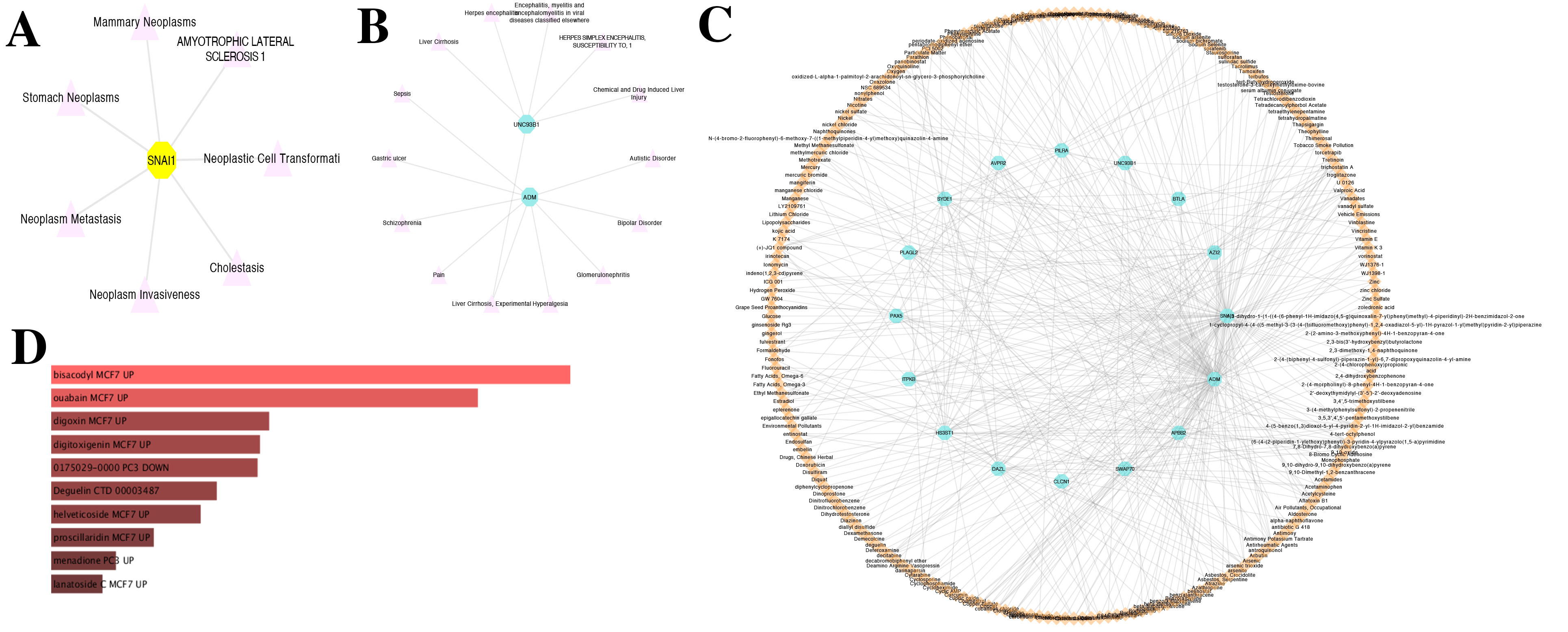

Supplement: Supplementary file 4 — Figure S4. Regulation factors of feature genes. [file 41537_2026_744_MOESM4_ESM.jpg]

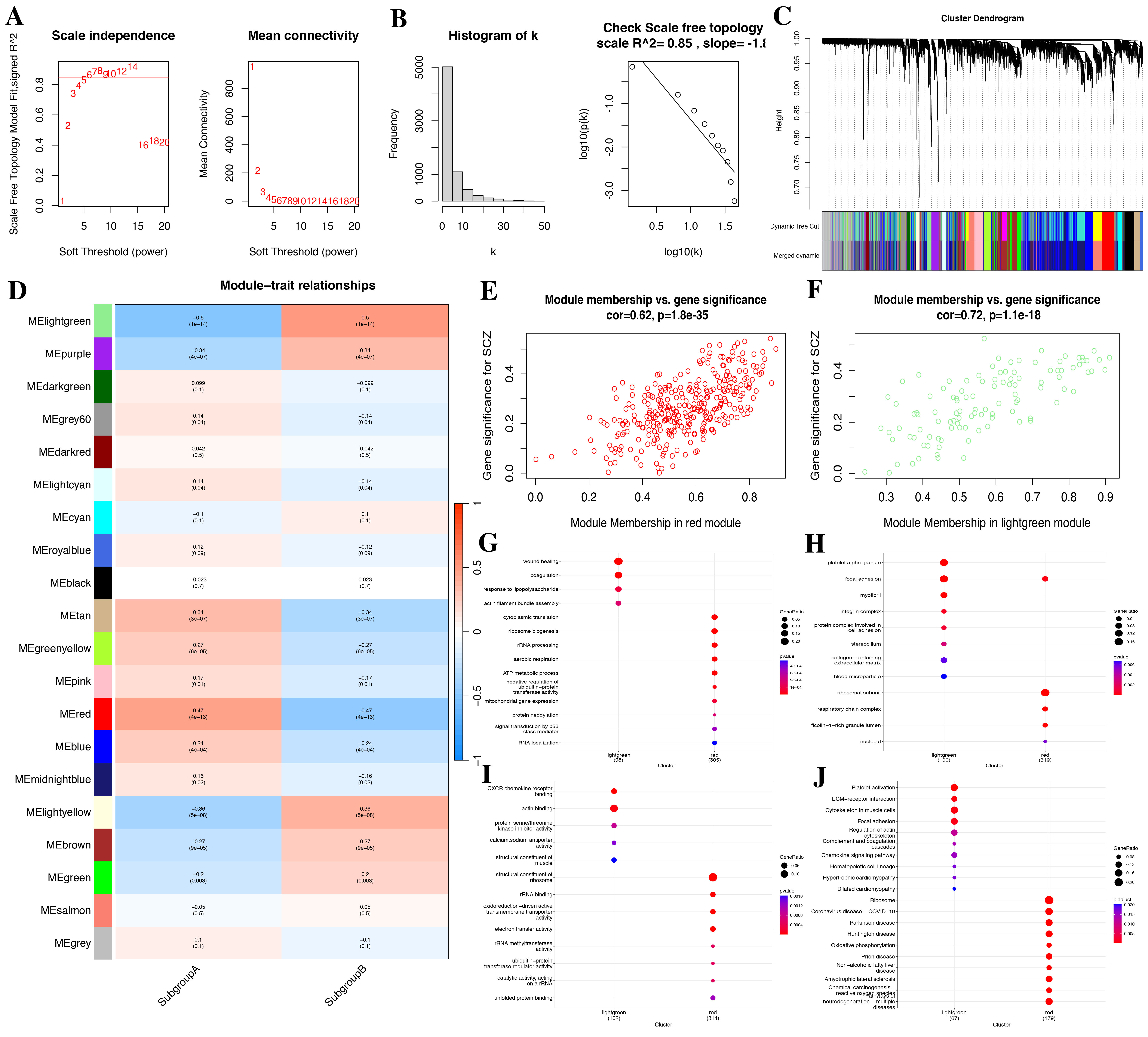

Supplement: Supplementary file 5 — Figure S5. Identification of hub genes between the two subgroups by the DEGs and WGCNA method. [file 41537_2026_744_MOESM5_ESM.jpg]

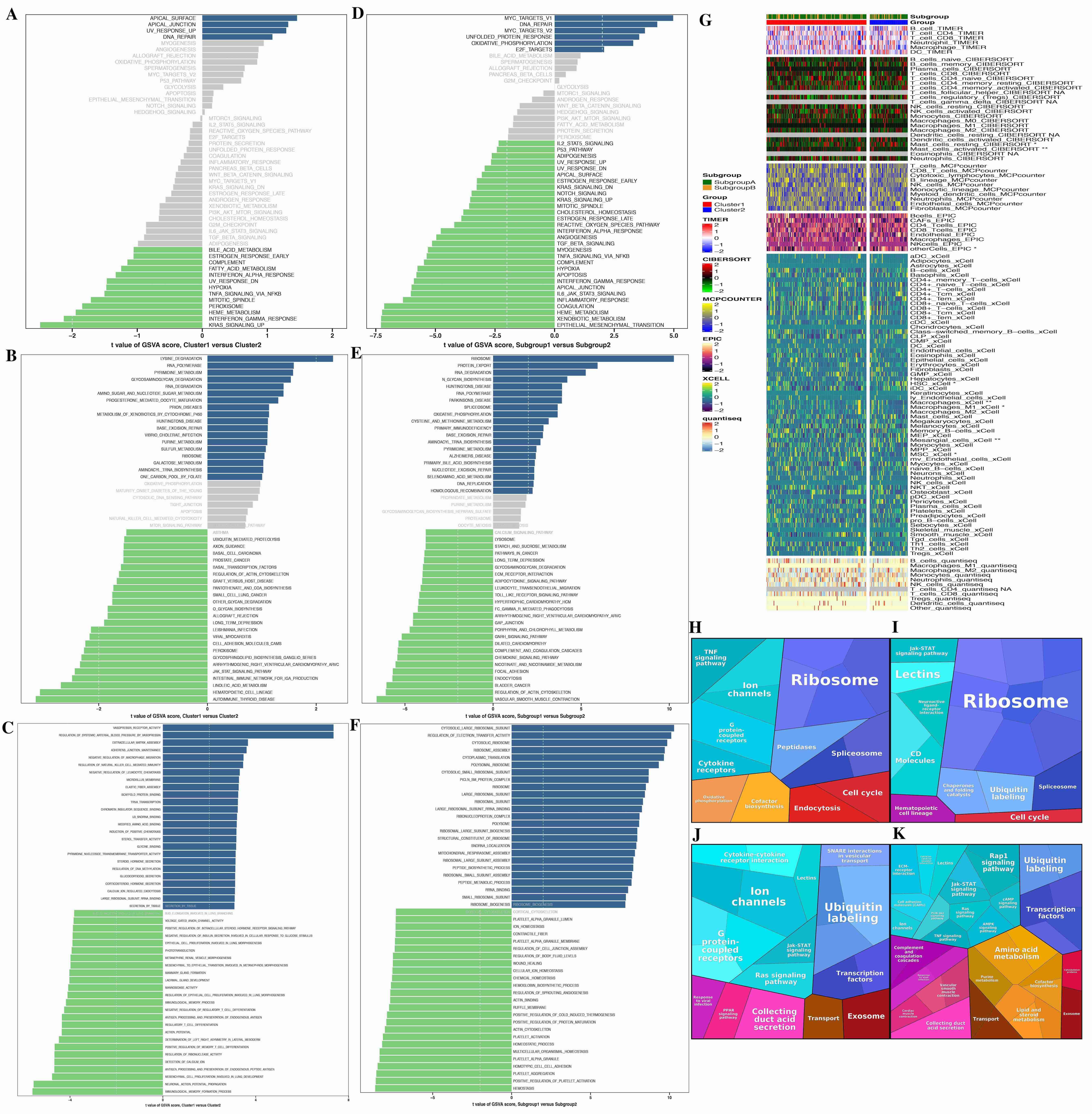

Supplement: Supplementary file 6 — Figure S6. Biological function and pathway, immune landscape and protein-level differences between the two subtypes. [file 41537_2026_744_MOESM6_ESM.jpg]

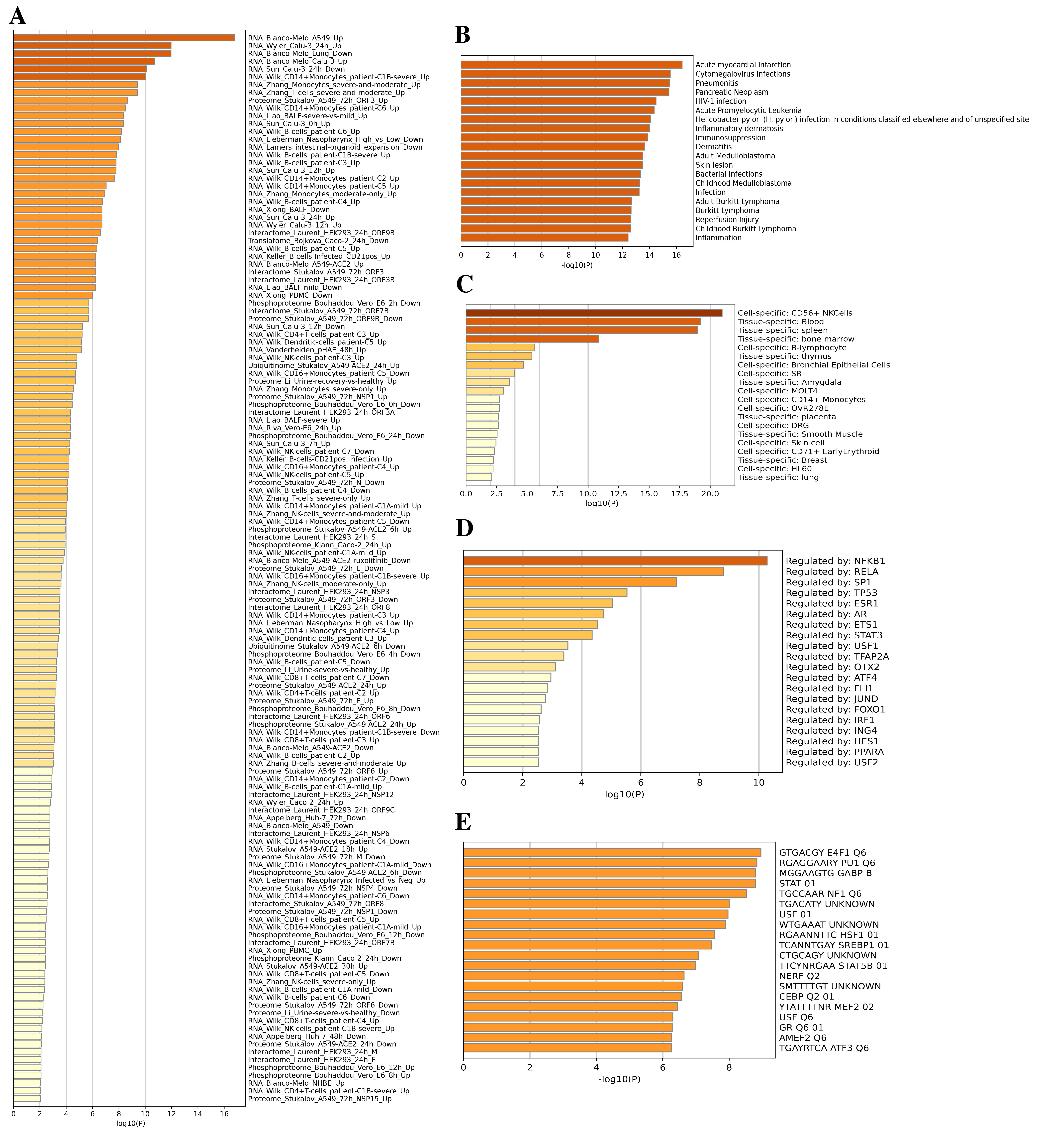

Supplement: Supplementary file 7 — Figure S7. Functional enrichment analysis by Metascape. [file 41537_2026_744_MOESM7_ESM.jpg]

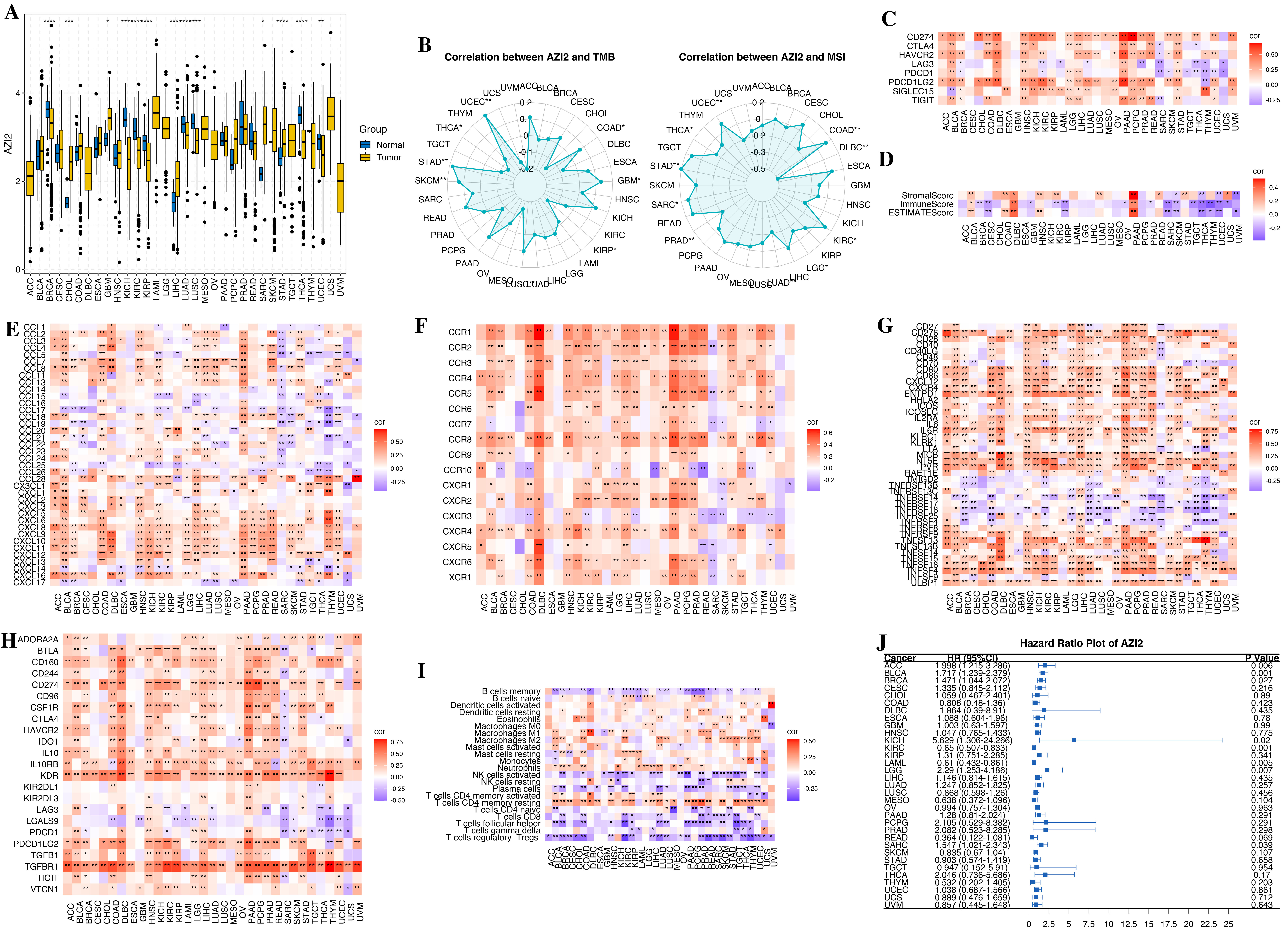

Supplement: Supplementary file 8 — Figure S8. Pan-cancer analysis of AZI2. [file 41537_2026_744_MOESM8_ESM.jpg]

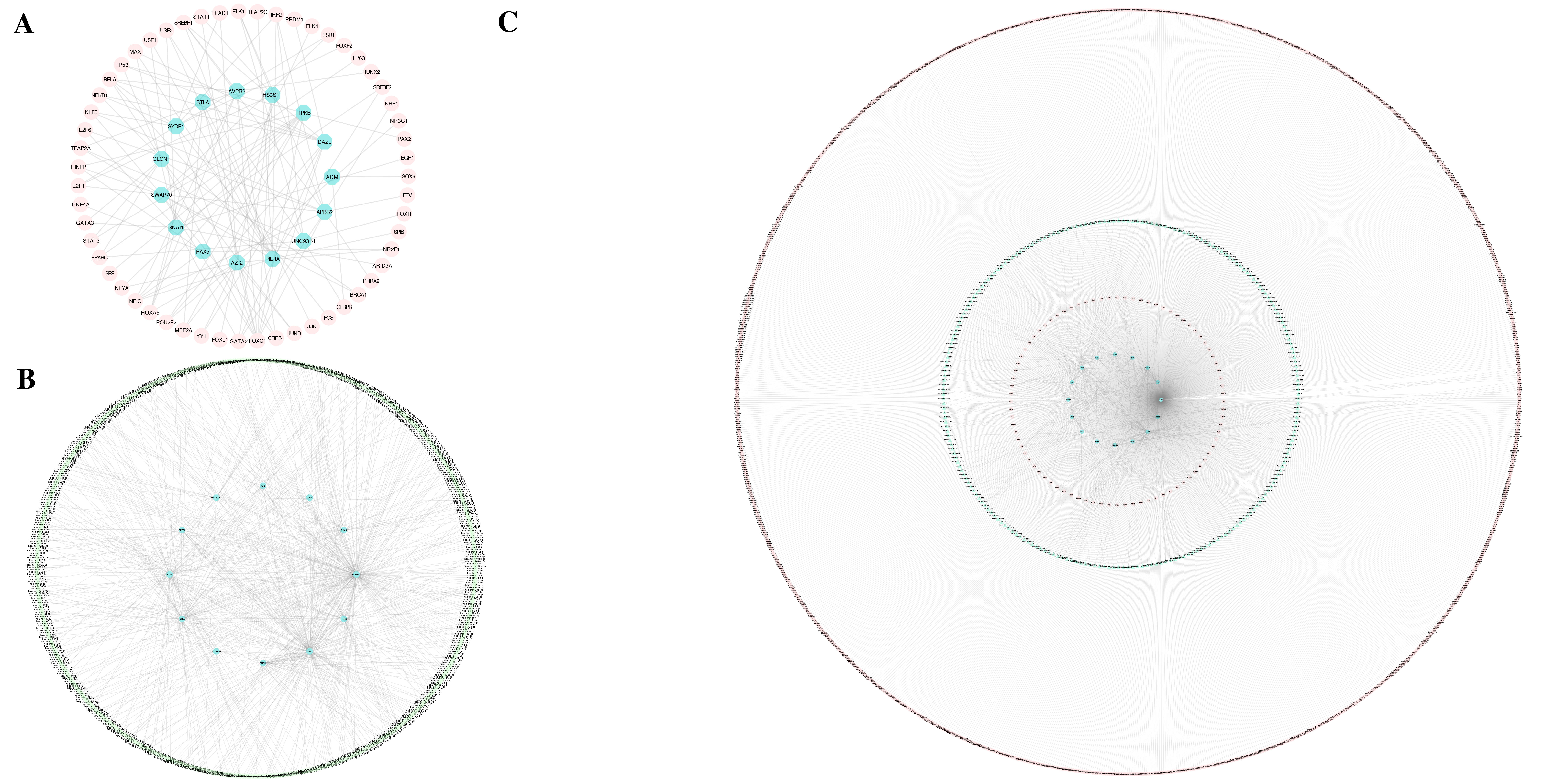

Supplement: Supplementary file 9 — Figure S9. Regulation factors. [file 41537_2026_744_MOESM9_ESM.jpg]
