# Supplementary material for: Machine learning-based predictive models and subtypes patterns in peripheral blood of schizophrenia based on a machine learning computational framework
Source: Schizophrenia (Heidelb). 2026 Mar 24;12(1):46. doi: 10.1038/s41537-026-00744-z (PMC13187130; doi:10.1038/s41537-026-00744-z)
Supplement: Supplementary file 11 — Supplemental files legends [file 41537_2026_744_MOESM11_ESM.docx]

Figure S1. 78 overlapping genes and functional enrichment analysis.

A, Overlapping genes intersected by DEGs and Key module genes. B, Heatmap of the top 20 enriched GO terms for 78 genes by Metascape. C, Network of enriched terms colored by cluster ID, where nodes that share the same cluster ID are typically close to each other. D, Enrichment analysis result in COVID. E, Enrichment analysis result in DisGeNet. F, Enrichment analysis result in Cell Type Signatures. G, Enrichment analysis result in PaGenBase. H, Enrichment analysis result in TRRUST.

Figure S2. A diagnostic signature of SCZ was developed.

A, The ROC values for diagnosing SCZ using the 133 algorithms on the eight sets are shown in a heatmap. B-Q, Nomogram displaying the predicted risk for SCZ based on the diagnostic signature and Calibration curve showing the predicted performance in nine sets. B-C, Train. D-E, Test. F-G, All samples-Meta. H-I, GSE18312. J-K, GSE27383. L-M, GSE38485, N-O, GSE54913. P-Q, GSE165604. R-S, ROC values and DCA of Nomogram model with the diagnostic signature on the nine sets displaying the predicted risk and showing the clinical benefits for SCZ.

Figure S3. Enrichment pathway analysis.

A-P The sixteen model genes were analyzed using GSVA.

Figure S4. Regulation factors of feature genes.

A-B, Gene-diseases. C, Protein-chemical interaction network by CTD. D, Bar chart of the Top10 drugs that associated with model genes by DSigDB.

Figure S5. Identification of hub genes between the two subgroups by the DEGs and WGCNA method.

A, Soft Threshold. B, Free topology scale R2. C, Cluster dendrogram of all genes. D, Correlation of modules and disease status. E-F, Scatterplots of GS for disease status versus module membership (MM) in key modules. G, GO terms of biological process (BP). H, GO terms of cellular component (CC). I, GO terms of molecular function (MF). J, KEGG pathways.

Figure S6. Biological function and pathway, immune landscape and protein-level differences between the two subtypes.

A-F, GSVA enrichment pathway based on the HALLMARK, KEGG and biological process pathway between two clusters (A-C), and between two subgroups (D-F). G, Immune landscape by six algorithms. D-E, ProteoMaps of upregulated and downregulated genes between two clusters (H, J) and between two subgroups (I, K)

Figure S7. Functional enrichment analysis by Metascape.

A, Enrichment analysis result in COVID. B, Enrichment analysis result in DisGeNet. C, Enrichment analysis result in PaGenBase. D, Enrichment analysis result in TRRUST. E, Enrichment analysis result in Transcription Factor Targets.

Figure S8. Pan-cancer analysis of AZI2.

A, Differential analysis. B, TMB and MSI analysis. C-H, Correlation analysis, including immune score (C), immunological checkpoints (D), chemokine (E) and chemokine receptors (F), immune stimulators (G) and immune inhibitors (H). I, Survival analysis.

Figure S9. Regulation factors.

A, TFs. B, miRNAs. C, TFs-miRNAs-Genes co-regulation network.

Table S1. Suggested top 10 chemicals for SCZ.

Table S2. Suggested top 10 drugs for SCZ.

Table S3: Detailed information of microarray datasets from GEO.

Table S4. Functional and pathway Enrichment of DEGs in schizophrenia.

Table S5. Key modules and key module genes.

Table S6. 78 overlapping genes intersected by DEGs and Key module genes.

Table S7: The classification performance metrics for diagnosing SCZ using the 133 ML algorithms on the eight sets.

Table S8: Features of the 133 ML algorithms for diagnosing SCZ.

Table S9: Functional and pathway Enrichment of 16 features selected by ML algorithms in schizophrenia.

Table S10: GSEA of DEGs among Clusters and Subgroups.

Table S11: Key Module and hub genes of Subgroups.
